# Peripheral Ulcerative Keratitis as a Manifestation of Drug-Induced Cicatrizing Conjunctivitis

**DOI:** 10.7759/cureus.34115

**Published:** 2023-01-23

**Authors:** Aniruddha Soni, Khushi Shah, Mansi Shah, Jayesh Vazirani

**Affiliations:** 1 Medicine, Jawaharlal Nehru Medical College, Wardha, IND; 2 Ophthalmology, Center for Excellence in Cornea and Ocular Surface Disorders, Excel Eye Care, Ahmedabad, IND

**Keywords:** dicc, puk, chronic cicatrizing conjunctivitis, anti glaucoma medications, drug toxicity, drug induced cicatrizing conjunctivitis, peripheral ulcerative keratitis

## Abstract

Ocular surface drug toxicity due to the long-term use of topical medication is a commonly overlooked cause of chronic conjunctival inflammation. A variety of eye drops, including but not limited to anti-glaucoma medications can cause drug-induced cicatrizing conjunctivitis. The classical descriptions of this condition include inflammation and scarring involving the eyelids, puncta, and conjunctiva. Herein, we present a case with bilateral peripheral ulcerative keratitis as a manifestation of drug-induced cicatrizing conjunctivitis.

## Introduction

Chronic cicatrizing conjunctivitis (CCC) can be a primary ocular disease without systemic involvement or a part of multi-system disorders that cause conjunctival scarring, potentially leading to blinding sequelae. Signs include eyelid abnormalities like entropion, trichiasis & distichiasis, caruncular and/or subconjunctival fibrosis progressing to forniceal foreshortening and symblepharon formation, limbal stem cell deficiency, and even dermalization of the ocular surface [1]. An important cause of CCC is ocular surface drug toxicity, commonly due to the use of anti-glaucoma medications (AGM) [2]. Herein, we present an unusual clinical manifestation of drug-induced cicatrizing conjunctivitis (DICC).

## Case presentation

**Figure 1:**
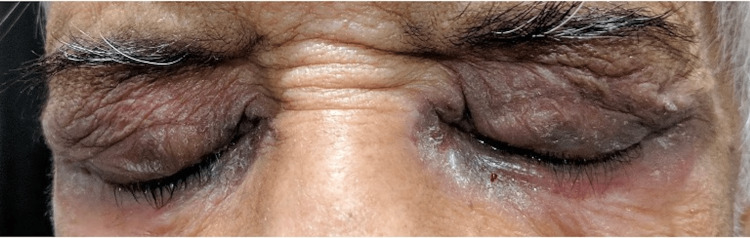
External photograph showing excoriation and pigmentation of eyelid skin in both eyes

**Figure 2:**
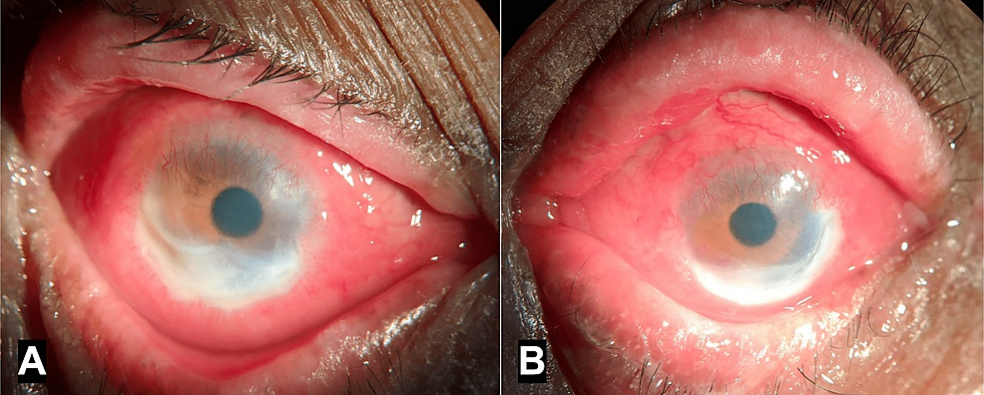
Diffuse illumination photographs showing conjunctival congestion with crescentic peripheral corneal ulcers in the right eye (A) and left eye (B)

A 78-year-old man with a history of well-controlled type-2 diabetes and hypertension presented with complaints of redness & discharge in both eyes for three months and diminished vision for a few years. He was using moxifloxacin, amikacin, and atropine eye drops in both eyes, and mometasone furoate + fusidic acid ointment on the eyelid skin for two weeks. He was earlier diagnosed to have glaucoma in both eyes, and was using latanoprost, timolol, and brinzolamide eye drops for a few months. He had undergone cataract surgery in both eyes many years ago. The visual acuity in the right eye was hand motions close to face (HMCF), with projection of rays accurate in the nasal and temporal quadrants. The left eye had perception of light (PL), with projection of rays accurate only in the inferior quadrant. The intraocular pressure could not be assessed accurately. External examination showed excoriation and pigmentation of the upper and lower eyelid skin in both eyes (Figure 1). Slit lamp examination revealed thickened eyelid margins with distorted mucocutaneous junctions and no visible meibomian gland openings, occluded puncta, bilateral severe conjunctival and ciliary congestion, scarring in the inferior fornices and peripheral superficial corneal vascularisation in both eyes. Peripheral crescentic corneal epithelial defects with stromal infiltrates and >70% thinning were visible in the right eye from 4-10 o’clock position, and in the left eye from 2-8 o’clock position (Figure 2). The anterior chamber was quiet in each eye, and the posterior chamber intraocular lenses were visible. Posterior segment examination showed attached retina with near total cupping and disc pallor in both eyes.

A provisional diagnosis of bilateral peripheral ulcerative keratitis (PUK) with pseudophakia and glaucoma was made. Systemic evaluation and laboratory investigations were negative for systemic autoimmune diseases. Based on the history, temporal association of symptoms with the use of eye drops, and presence of conjunctival inflammation and scarring, a diagnosis of PUK associated with DICC was considered to be most plausible.

**Figure 3:**
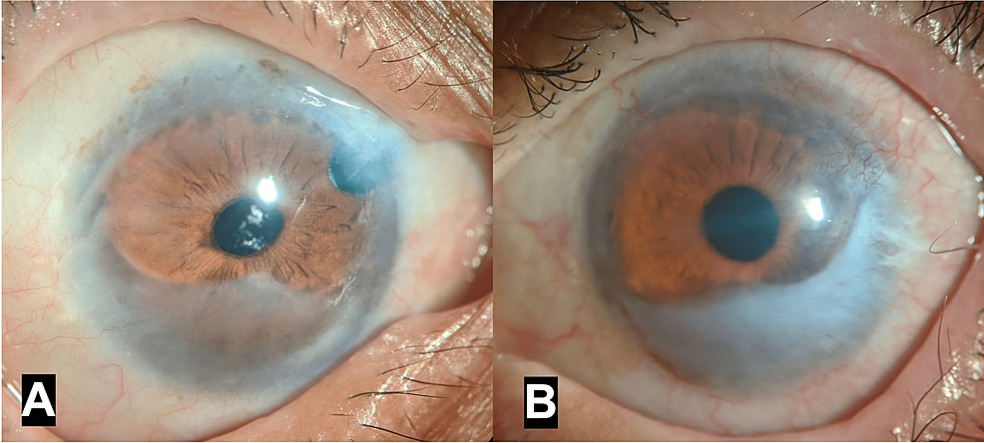
Diffuse illumination photographs showing a stable ocular surface with corneal scars in the right eye (A) and left eye (B) at the last follow-up visit

All eye drops were discontinued. Oral acetazolamide was started for controlling intraocular pressure. Prednisolone acetate 1% eye drops were started six times per day in each eye and tapered over six weeks. Lateral paramedian permanent tarsorrhaphy was done in each eye. As the ocular surface inflammation decreased and corneal epithelium healed, preservative-free travoprost eye drops were started. Six months later, a trabeculectomy was performed in the right eye. The patient achieved a best-corrected visual acuity of 6/18 in the right eye after surgery, with good control of intraocular pressure. This was maintained till the last follow-up visit 18 months after the initial presentation (Figure 3). The left eye retained a visual acuity of PL, and no further intervention was advised in view of advanced optic atrophy. 

## Discussion

Peripheral ulcerative keratitis is an inflammatory disease of the juxtalimbal cornea, associated with a crescent-shaped corneal epithelial defect, stromal thinning, and inflammatory corneal infiltration. Autoimmune collagen vascular diseases such as rheumatoid arthritis, granulomatosis with polyangiitis, and systemic lupus erythematosus are well-known systemic associations in cases of PUK [3]. These cases are typically treated with systemic corticosteroids and immunosuppressive therapy. Our case had morphological features indistinguishable from cases of PUK that are typically associated with systemic autoimmune disease. The absence of clinical or laboratory evidence of autoimmune disease, temporal association of symptoms with the use of AGM, and the resolution of PUK on discontinuing eye drops clearly indicates ocular surface drug toxicity as the underlying cause. 

Drug-induced cicatrizing conjunctivitis can be defined as a disease in which conjunctival cicatrization develops as a response to the chronic use of inciting topical and, rarely, systemic medications. The fibrotic changes in DICC are usually concentrated in the inferior fornix and medial canthi, indicating damage to the areas of the ocular surface that come in maximal contact with the medication. It occurs after long-term use of topical ocular medications, most commonly AGM. The pathogenesis may include chronic cicatrizing conjunctivitis, type I or type IV hypersensitivity, and non-specific irritative/toxic conjunctivitis. Preservatives used in AGM such as benzalkonium chloride as well as the drug itself may be responsible for these effects. The management of DICC involves discontinuing the inciting topical medications. Ocular surface inflammation may subside rapidly on drug withdrawal but may benefit from treatment with topical steroids. In a recently published case series, management modalities consisted of stopping topical AGM in all patients, substituting with preservative-free or oral AGM in 76.2% of eyes, and a short course of topical steroids in 23.8% of eyes. Ten of 42 eyes with uncontrolled IOP despite oral AGM required surgical interventions such as trabeculectomy, Baerveldt tube (shunt) surgery, and transscleral laser photocoagulation [4].

Inflammatory changes in the eyelids, puncta, and conjunctiva are commonly seen in cases of DICC, with corneal signs usually restricted to punctate epithelial erosions. Peripheral ulcerative keratitis as a manifestation of DICC has not yet been reported. This case illustrates that DICC may present with corneal lesions that resemble autoimmune PUK. The inferior location of the corneal lesions indicates that corneal epithelial breakdown with progressive stromal infiltration and thinning occurred in the area of maximal contact with topical medication. The lesions healed rapidly on withdrawing the offending drugs. Long-term ocular surface stability was maintained without any recurrence of PUK, further confirming a local rather than a systemic cause for the corneal ulceration.

## Conclusions

Peripheral ulcerative keratitis can be a manifestation of drug-induced cicatrizing conjunctivitis. The presence of chronic conjunctival inflammation with PUK in a patient on long-term topical medication, especially AGM, should alert the clinician to this possibility. In the absence of associated autoimmune disease, it may be prudent to withhold systemic corticosteroids and immunosuppressive therapy. As a first step, topical medication should be discontinued. Further management includes using topical steroids to control the surface inflammation and surgery for IOP control if necessary.
